# Corticosteroid therapy in critically ill patients with COVID-19: a multicenter, retrospective study

**DOI:** 10.1186/s13054-020-03429-w

**Published:** 2020-12-18

**Authors:** Yiming Li, Qinghe Meng, Xin Rao, Binbin Wang, Xingguo Zhang, Fang Dong, Tao Yu, Zhongyi Li, Huibin Feng, Jinpeng Zhang, Xiangyang Chen, Hunian Li, Yi Cheng, Xiaoyang Hong, Xiang Wang, Yimei Yin, Zhongheng Zhang, Dawei Wang

**Affiliations:** 1 Department of Critical Care Medicine, Zhongnan Hospital of Wuhan University, Wuhan, Hubei China; 2 Department of Surgery, SUNY Upstate Medical University, Syracuse, NY USA; 3 Department of Critical Care Medicine, Xishui People’s Hospital, Huanggang, Hubei China; 4 Wuhan Third Hospital & Tongren Hospital of Wuhan University, Wuhan, Hubei China; 5 Department of Infectious Disease, Weihai Municipal Hospital, Weihai, Shandong China; 6 Department of Critical Care Medicine, Wuhan Ninth Hospital, Wuhan, Hubei China; 7 Department of Critical Care Medicine, Huangshi Central Hospital, Huangshi, Hubei China; 8 Department of Critical Care Medicine, Huanggang Central Hospital, Huanggang, Hubei China; 9 Department of Critical Care Medicine, Tuanfeng People’s Hospital, Huanggang, Hubei China; 10 Department of Critical Care Medicine, Shiyan People’s Hospital, Shiyan, Hubei China; 11 Department of Critical Care Medicine, Huangshi Aikang Hospital, Huangshi, Hubei China; 12 Department of Critical Care Medicine, Huangmei People’s Hospital, Huanggang, Hubei China; 13 Department of Critical Care Medicine, Dongfeng Motor General Hospital, Shiyan, Hubei China; 14 Department of Ultrasound Medicine, Zhongnan Hospital of Wuhan University, Wuhan, Hubei China; 15 Department of Emergency Medicine, Sir Run Run Shaw Hospital, Zhejiang University School of Medicine, Hangzhou, Zhejiang China

**Keywords:** COVID-19, Corticosteroid, Marginal structural modeling

## Abstract

**Background:**

Corticoid therapy has been recommended in the treatment of critically ill patients with COVID-19, yet its efficacy is currently still under evaluation. We investigated the effect of corticosteroid treatment on 90-day mortality and SARS-CoV-2 RNA clearance in severe patients with COVID-19.

**Methods:**

294 critically ill patients with COVID-19 were recruited between December 30, 2019 and February 19, 2020. Logistic regression, Cox proportional-hazards model and marginal structural modeling (MSM) were applied to evaluate the associations between corticosteroid use and corresponding outcome variables.

**Results:**

Out of the 294 critically ill patients affected by COVID-19, 183 (62.2%) received corticosteroids, with methylprednisolone as the most frequently administered corticosteroid (175 accounting for 96%). Of those treated with corticosteroids, 69.4% received corticosteroid prior to ICU admission. When adjustments and subgroup analysis were not performed, no significant associations between corticosteroids use and 90-day mortality or SARS-CoV-2 RNA clearance were found. However, when stratified analysis based on corticosteroid initiation time was performed, there was a significant correlation between corticosteroid use (≤ 3 day after ICU admission) and 90-day mortality (logistic regression adjusted for baseline: OR 4.49, 95% CI 1.17–17.25, *p* = 0.025; Cox adjusted for baseline and time varying variables: HR 3.89, 95% CI 1.94–7.82, *p* < 0.001; MSM adjusted for baseline and time-dependent variants: OR 2.32, 95% CI 1.16–4.65, *p* = 0.017). No association was found between corticosteroid use and SARS-CoV-2 RNA clearance even after stratification by initiation time of corticosteroids and adjustments for confounding factors (corticosteroids use ≤ 3 days initiation vs no corticosteroids use) using MSM were performed.

**Conclusions:**

Early initiation of corticosteroid use (≤ 3 days after ICU admission) was associated with an increased 90-day mortality. Early use of methylprednisolone in the ICU is therefore not recommended in patients with severe COVID-19.

## Background

Coronavirus disease 2019 (COVID-19) is caused by SARS-CoV-2 (severe acute respiratory syndrome coronavirus 2), which has been at the center of an ongoing global pandemic [1]. Corticoid therapy is currently a key treatment regimen and recommended specifically for critically ill patients in the intensive care unit (ICU) [2]. However, the effect of corticoid treatment for COVID-19 patients is still under evaluation. For example, one study showed that in hospitalized patients with COVID-19, the use of dexamethasone reduced 28-day mortality in both patients who received invasive mechanical ventilation or oxygen alone [3]. Another systemic review and meta-analysis showed that dexamethasone reduced mortality and the use of mechanical ventilation in COVID-19 patients compared to those on standard care [4]. A third study demonstrated that methylprednisolone administration reduced the risk of death in COVID-19 patients complicated with acute respiratory distress syndrome (ARDS), although there was no adjustment for immortal time bias and indication bias by time-varying confounding variables [5]. In contrast, in a randomized trial from Brazil including 393 COVID-19 patients indicated no significance difference in 28-day mortality rates between administration of methylprednisolone and placebo [6].

Due to uncertainty regarding efficacy of corticosteroid in the treatment of COVID-19, it is imperative to evaluate its role in the management of COVID-19. Immortal time bias and indication bias should be adjusted when evaluating the role of corticosteroid for COVID-19 patients in observational cohort studies. Immortal time bias refers to the requirement that patients survive long enough to receive the intervention of interest, leading to a potential incorrect overestimation of a positive treatment effect [7]. Indication bias from time-varying confounding variables refers to having an association related to the indication of the intervention that evolves throughout the course of an illness [8]. MSM allows proper adjustment for immortal time bias and indication bias. In this retrospective study, we investigated the therapeutic effect of corticosteroids for the patients with COVID-19 using multivariate analyses. Logistic regression, Cox proportional-hazards model (Cox) and marginal structural modeling (MSM) were utilized to explore the relationship between the initiation of corticosteroid therapy and 90-day mortality as well as SARS-CoV-2 RNA clearance. Importantly, baseline and time varying confounding factors were adjusted, with MSM allowing for proper adjustment of time-dependent confounding variables.

## Methods

### Study design and participants

We retrospectively studied all critically ill patients with COVID-19 admitted to 10 Intensive Care Units (ICUs) in Hubei province between December 30, 2019 and February 19, 2020. The data was censored on April 9, 2020. All patients were consecutive. Those receiving chronic corticosteroid therapy before the onset of critical illness were excluded. COVID-19 was diagnosed according to World Health Organization interim guidance. Critically ill patients were defined as those admitted to the ICU who required mechanical ventilation or had a fraction of inspired oxygen of at least 60%. This study was approved by the institutional ethics board of the participating medical centers (NO. 2020045).

### Data collection

Data was extracted from the medical records of patients, reviewed and verified by the research team from the Department of Critical Care Medicine, Zhongnan Hospital of Wuhan University. Extracted data included epidemic data, demographic data, comorbidities, complications, treatments and time course of illness. Laboratory findings and respiratory mechanic variables on day 1 of ICU admission were also collected. Severity of illness was assessed using the Acute Physiology and Chronic Health Evaluation (APACHE) II score and organ dysfunction was assessed using the Sequential Organ Failure Assessment (SOFA) score, both of which were recorded on day 1 of ICU admission. The primary outcome was determined as 90-day mortality after ICU admission and secondary outcome as duration of SARS-CoV-2 RNA clearance.

ARDS was diagnosed according to the Berlin Definition and acute kidney injury (AKI) was diagnosed according to the Kidney Disease: Improving Global Outcomes (KDIGO) clinical practice guidelines [9, 10]. Acute cardiac injury was defined as blood levels of hypersensitive troponin I above the upper reference limit (> 26.2 pg/ml). Duration of SARS-CoV-2 RNA clearance was defined as the time from ICU admission until the occurrence of two negative Nucleic-acid Amplification tests (NAAT) without a positive test afterward. Corticosteroid therapy was defined as the use of systemic corticosteroids when dosage equivalent to at least 60 mg of hydrocortisone per day [11]. The type, daily dose, and duration of corticosteroid administration were collected. All preparations were converted to hydrocortisone-equivalent doses (methylprednisolone 1:5, dexamethasone 1:25, prednisolone 1:4).

### Statistical analysis

Categorical variables were presented as frequency rates and percentages. Continuous variables were described using mean and standard deviations or medians and IQR values according to the distribution of the data. We compared characteristics of patients who received or not received corticosteroid therapy using chi-square test or Fisher’s exact test for categorical variables and student *t*-test or Mann–Whitney test for continuous variables, as appropriate. Subgroup analyses were performed by stratification to (1) high-dose corticosteroid therapy (≥ 300 mg of hydrocortisone equivalent per day) and low-dose corticosteroid therapy (< 300 mg of hydrocortisone equivalent per day) versus no treatment with corticosteroids, respectively; (2) corticosteroid therapy initiated in the first 3 days of ICU admission versus no corticosteroid therapy; (3) corticosteroid therapy started after Day 3 versus no corticosteroid therapy.

All statistical analyses were performed using R Project. A *p* value less than 0.05 was considered statistically significant. We calculated the a priori sample size using online software (A-priori Sample Size Calculator for Multiple Regression: https://www.danielsoper.com/statcalc/calculator.aspx?id=1). Totally, 14 variables were selected in our study. We assumed that anticipated effect size was 15%, desired statistical power was 80%, alpha level (type I error) was 0.05 [12–14]. The calculated sample size was 135. In current study, 294 patients were included (survivor: 148, non-survivor: 146). We tested the associations of corticosteroid therapy and 90-day mortality or SARS-CoV-2 RNA clearance using three approaches: logistic regression, Cox and MSM.

### Logistic regression

Logistic regression adjusted for baseline differences only. We included in the multivariable model a priori–decided baseline variables of clinical interest and all significant variables at the univariable level (*p* < 0.2).

### Cox proportional hazards regression

Cox was used to adjust for baseline differences and immortal time bias. To examine outcomes as a time to event (i.e., death), we performed a Cox adjusting for the same above-mentioned baseline covariates with corticosteroid therapy as a time-varying covariate.

### Marginal structural model

MSM was used to adjust for baseline differences and time varying confounders (indication bias and immortal time bias). Time-varying variables were determined as PaO_2_/FiO_2_, ventilation status and SOFA scores on the day of corticosteroid therapy initiation. Refining marginal structural models involves the calculation of two weights for each observation: treatment selection weight and censoring weight. The treatment selection weight at time k is a ratio of two weights. The numerator is the product of probabilities that a patient receives his observed treatment at time k, given the baseline variable. The denominator is calculated similarly by incorporating the time-varying variable (SOFA on the day, SOFA on the previous day, mode of ventilation on the day and mode of ventilation on the previous day). The weights are recalibrated until the first day of corticosteroid therapy and kept constant afterward. Since SOFA scores were recorded on Days 1, 3, 7, 14, and 28, we imputed missing values for the remaining days using the following rules: (a) If ICU length of stay (LOS) ≥ 28, we used linear interpolation for each of the intervals 4–6 days, 8–13 days and 15–27 days. (b) If 14 ≤ ICU LOS ≤ 27, we used linear interpolation for 4–6 days and 8–13 days and the Last-Observation-Carried-Forward method from day 15 onwards. (c) Missing values are managed similarly for ICU LOS less than 14 days. Ventilation status was recorded also on Days 1, 3, 7, 14, and 28. We imputed the mode of ventilation between these days using the Last-Observation-Carried-Forward method.

The same approach was used to calculate the censoring weight for early patient dropout. Patients were considered censored at hospital discharge or at Day 90, and the weights for censoring are calculated as the ratio of a subject’s probability of remaining uncensored up to day *k*. The final weight for each observation was obtained by multiplying the treatment selection weights and the censoring weights. We used weight truncation to manage outliers; weights greater than the 95th percentile value were fixed at the 95th percentile value, and weight less than the 5th percentile value were fixed at the 5th percentile value. This process continued until the mean of weight was close to 1. The time-varying intercept was modeled by a smoothing function of time, using restricted cubic splines with five knots for days from beginning of follow-up day. For the analysis of the associations of corticosteroid therapy and SARS-CoV-2 RNA clearance, patients were censored if they never cleared SARS-CoV-2 RNA or if they were discharged alive from the hospital before clearing SARS-CoV-2 RNA.

## Results

### Patient characteristic

Data was collected from critically ill COVID-19 patients who admitted to ten participating hospitals between December 30, 2019 and February 19, 2020. Within the study period, two patients were excluded due to long-term treatment with glucocorticoids prior to contraction of COVID-19, and seven cases were excluded due to missing outcome variables. In total, 294 patients with COVID-19 were included in this study. 183 of 294 patients (62.2%) received corticosteroid therapy (Table 1). Immunosuppression was more common in non-corticosteroid group than in corticosteroid group. Troponin I (TnI), bilirubin and SOFA scores of corticosteroid group were higher than those of non-corticosteroid group. Lymphopenia was more obvious in corticosteroid group than in non-corticosteroid group. Respiratory failure in patients receiving corticosteroid treatment was more severe than that in patients not receiving corticosteroid treatment, resulting in a lower PaO_2_/FiO_2_ value, higher percentage of mechanical ventilation, longer ventilation duration, and higher frequency of ARDS. Secondary infections are more likely to occur in the corticosteroid group during ICU stay. However, there was no significant difference in crude 90-day mortality between patients treated with corticosteroid and those not treated with corticosteroid (97 of 183 [53%] vs 49 of 111 [44%]; *p* = 0.18). 90-day survivors in the corticosteroid-treated group took longer to clear RNA than those in the non-corticosteroid group (10 [5, 17.5] vs 7 [1, 15]; *p* = 0.049).

**Table 1:** Clinical characteristic of patients with COVID-19 in the corticosteroid therapy and no corticosteroid therapy groups

	Total, *n* = 294	Corticosteroids, *n* = 183	No corticosteroids, *n* = 111	*p* value
Age, years	66 (56–75)	65 (56, 73)	67 (56, 78.5)	0.14
Male Sex	197 (67%)	151 (68%)	73 (66%)	0.91
Comorbidity				
Hypertension	134 (46%)	82 (45%)	52 (47%)	0.83
Diabetes	80 (27%)	53 (29%)	27 (24%)	0.47
Heart disease	68 (23%)	40 (22%)	28 (25%)	0.6
Chronic lung disease	36 (12%)	28 (15%)	8 (7%)	0.062
Cerebrovascular disease	31 (11%)	15 (8%)	16 (14%)	0.14
Immunosuppression	14 (5%)	7 (3%)	7 (10%)	0.047
PEEP,cmH2O	9 (6, 10)	8 (6, 10)	10 (6, 11)	0.56
PaO_2_/FiO_2_, mmHg	138 (86.5–204)	119.5 (80, 181.5)	176 (108, 260)	< 0.001
Platequ pressure, cmH_2_O	25 (21–26)	25 (22.5, 28)	25 (21, 26)	0.057
Mean arterial pressure, mmHg	90 (80–97)	90 (81, 97)	90 (80, 97)	0.7
Creatinine, μmol/L	76 (61.7–101.2)	76.35 (63.7, 100.8)	72.65 (56.3, 103.4)	0.24
Elevated TnI,	95 (41%)	52 (35%)	43 (51%)	0.024
Bilirubin, μmol/L	22.2 (13.2–24)	23 (14.72, 24)	21 (10.9, 24)	0.028
Platelet count, × 10^9^/L	158 (114–207.3)	154 (113, 197.3)	168.1 (118.3, 229.8)	0.1
Lymphocyte percentage, %	6.2 (2.6–11.3)	5.4 (2.1, 10.11)	7.2 (3.8, 15.3)	0.006
APACHEII score	14.5 (11, 18)	15 (11, 18)	13 (11, 17)	0.35
SOFA score	4 (3–5.75)	4 (3, 6)	3 (2, 5)	0.005
Complication				
Shock	121 (42%)	83 (46%)	38 (35%)	0.076
ARDS	214 (74%)	146 (81)	68 (62%)	< 0.001
AKI	90 (31%)	61 (34%)	29 (26%)	0.24
Secondary infection,	44 (15%)	34 (19%)	10 (9%)	0.039
Respiratory support received				
Mechanical ventilation	230 (78.2%)	148 (80.8%)	82 (73.9%)	0.031
Prone	46 (16%)	31 (17%)	15 (14%)	0.56
ECMO	21 (7%)	17 (9%)	4 (4%)	0.1
Tracheotomy	23 (8%)	13 (7%)	10 (9%)	0.72
Requirement of CRRT	40 (14%)	26 (14%)	14 (13%)	0.83
Symptom onset to hospital admission, day	7(4–10)	7(4–10)	7 (3–14)	0.13
Symptom onset to ICU admission, day	11 (7–17)	11 (7.5, 16)	11 (6.5, 21)	0.91
Duration of ventilation, day	7 (0, 13)	8 (2, 15)	3 (0, 10)	< 0.001
ICU length of stay, day	13 (7–22)	13.5(8–23)	13 (5–20)	0.57
Hospital length of stay, day	20 (12–32)	21 (12.5–33)	17.5 (21–31)	0.34
90-day mortality	146 (50%)	97 (53%)	49 (44%)	0.18
Time to RNA clearance among 90-day survivors, day	9 (3–17)	10 (5, 17.5)	7 (1, 15)	0.049

Immunosuppression was defined as human immunodeficiency virus infection, malignancy, chemotherapy or organ transplantation. Laboratory and ventilator parameters on Day 1 of ICU admission were presented. Data presented as *n* (%) or median (*Q*1–*Q*3)

*PEEP* positive end-expiratory pressure, *Q* quartile, *SOFA* Sequential Organ Failure Assessment, *APACHEII* acute physiology and chronic health evaluation, *Tnl* Troponin I, *ECMO* extracorporeal membrane oxygenation, *ARDS* adult respiratory distress syndrome, *AKI* acute kidney injury

### Details of corticosteroid use

**Fig. 1:**
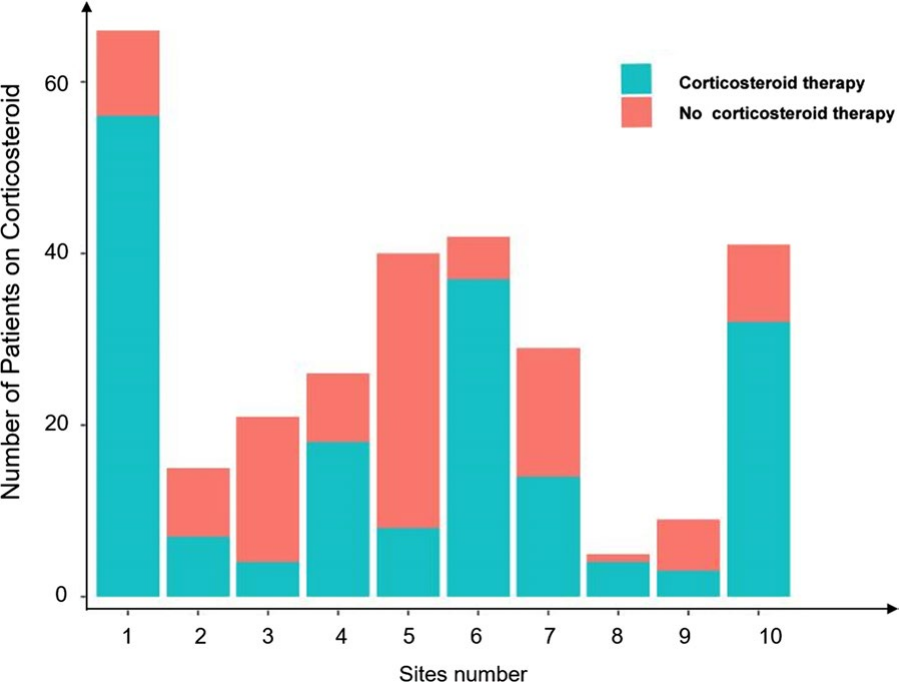
The distribution of patients from different ICU centers treated with or without corticosteroid

**Fig. 2:**
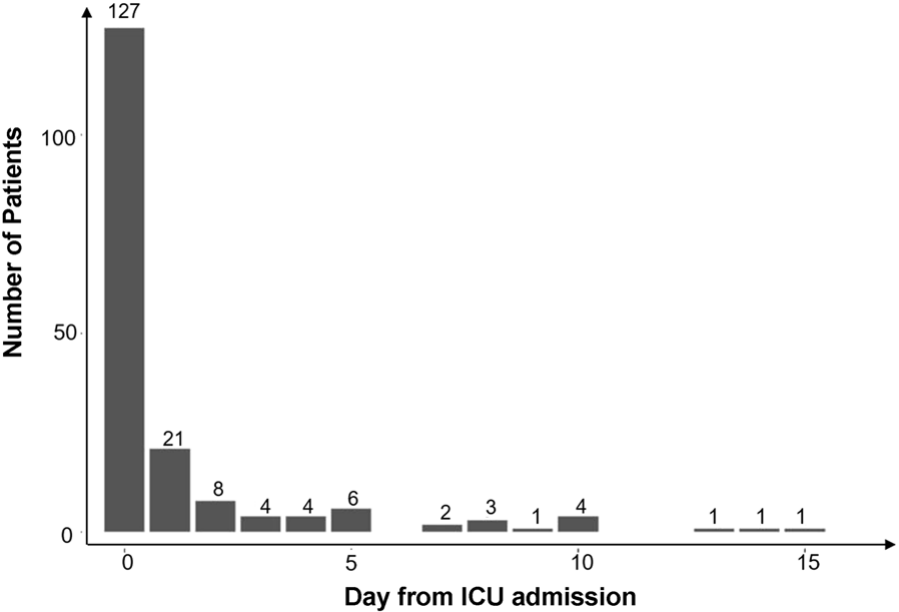
Time to corticosteroid therapy initiation from ICU admission. Day 0 includes patients who were already on corticosteroid therapy when admitted to the ICU

The distribution of patients with or without steroid therapy in 10 medical centers was shown in Fig. 1. The most widely-used steroid was methylprednisolone (Table 2), with 175 of 183 patients (96%) receiving methylprednisolone therapy and 11(6%) patients receiving hydrocortisone therapy. The median of Hydrocortisone equivalent per ICU day was 200 mg (IQR, 100–320.9) and the median duration of steroids treatment was 9 days (IQR, 5–14). The median of PaO_2_/FiO_2_ was 119 mmHg (IQR, 82–200) and SOFA score was 4 (IQR, 3–6) at corticosteroid initiation. 127 of 183 (69.4%) patients received corticosteroid treatment prior to ICU admission (Fig. 2). The time of corticosteroid initiation before ICU admission is shown in Additional file 1: Figure S1.

**Table 2:** Description of corticosteroid therapy among 183 critically ill patients with COVID-19

	Total, *n* = 183
Dexamethasone^a^	4 (2%)
Hydrocortisone^a^	11 (6%)
Methylprednisolone^a^	175 (96%)
Prednisone^a^	3 (2%)
Duration of corticosteroid treatment, day	9 (5–14)
Hydrocortisone equivalents per ICU day, mg/day	200 (100–320.9)
Duration between hospital admission and corticosteroid initiation, day	1 (0–5)
Duration between ICU admission and corticosteroid initiation, day	0 (0–2)
Duration between onset of ventilation and corticosteroid initiation, day	0 (0–4)
PaO_2_/FiO_2_ when corticosteroid initiation, mmHg	119 (82, 200)
SOFA score when corticosteroid initiation	4 (3, 6)

*Q* quartile, *SOFA* Sequential Organ Failure Assessment

^a^Percentages of dexamethasone, hydrocortisone, methylprednisolone and prednisone add to more than 100% because some patients received one or more formulation of corticosteroids. Data presented as *n* (%) or median (*Q*1–*Q*3)

### The association of corticosteroid use and 90-day mortality

Univariate analysis indicates that adverse outcomes are positively correlated with clinical condition at the time of ICU admission, including positive end-expiratory pressure (PEEP), PaO_2_/FiO_2_, APACHE II score and laboratory testing (creatinine, TnI, bilirubin and platelet count) (Additional file 2: Table S1). Non-survivors had more complications, including shock, ARDS and AKI. Variables were included in the multivariate analysis if *p* value ≤ 0.2. In our logistic regression, adjusted variables included age, hypertension, cardiovascular disease, PEEP, plateau pressure, mean arterial pressure, creatinine, TnI, lymphocyte percentage, bilirubin, platelet count, secondary infection and APACHE II score. After accounting for these adjustments, there was no association between corticosteroid therapy and 90-day mortality (OR, 0.15; 95% CI 0.00, 2.59; *p* = 0.22) (Table 3). There was also no significant association between corticosteroid treatment and 90-day mortality when analyzed using Cox (HR, 1.37; 95% CI 0.54, 3.47; *p* = 0.51) and MSM (OR, 1.37; 95% CI 0.93, 2.02; *p* = 0.11).

**Table 3:** Association between corticosteroid therapy and day-90 Mortality or SARS-CoV-2 RNA Clearance

	Day-90 mortality									SARS-CoV-2 RNA clearance					
	Logistic regression			Cox proportional hazards regression model			Marginal structural model			Cox proportional hazards regression model			Marginal structural model		
	OR	95%CI	*p* value	HR	95%CI	*p* value	OR	95%CI	*p* value	HR	95%CI	*p* value	OR	95%CI	*p* value
Corticosteroids versus no corticosteroids	0.15	0.00, 2.59	0.22	1.37	0.54, 3.47	0.51	1.37	0.93, 2.02	0.11	1.10	0.36, 3.37	0.86	0.85	0.63, 1.17	0.33
> 300 mg/day versus no corticosteroids^a^	NA	NA	NA	0.62	0.12, 3.26	0.57	1.01	0.62, 1.64	0.98	0.81	0.10, 6.38	0.84	0.67	0.44, 1.02	0.062
≤ 300 mg/day versus no Corticosteroids^a^	1.44	0.71, 2.96	0.31	1.42	0.94, 2.15	0.099	1.42	0.93, 2.17	0.1	0.91	0.22, 3.74	0.89	0.95	0.68, 1.34	0.78
> **3** day initiation versus no corticosteroids^b^	1.08	0.56, 2.12	0.81	1.06	0.70, 1.59	0.8	1.11	0.75, 1.66	0.6	0.91	0.65, 1.25	0.55	0.85	0.62, 1.17	0.33
≤ 3 day initiation versus no corticosteroids^b^	4.49	1.17, 17.25	0.025	3.89	1.94, 7.82	< 0.001	2.32	1.16, 4.65	0.017	0.26	0.13, 0.54	< 0.001	0.81	0.43, 1.52	0.5

*HR* hazard ratio, *OR* odds ratio, *CI* confidence interval, *MSM* marginal structural model, *NA* not available

^a^300 mg/day means hydrocortisone equivalents per ICU day

^b^> 3 day or ≤ 3 day means initiation of corticosteroids in more than 3 or less than 3 days after ICU admission

Sensitivity analysis was performed by comparing patients who were treated with different dosages of corticosteroids. MSM with inverse probability weighting analysis showed that both high and low dosage of corticosteroid therapy were not associated with mortality (corticosteroids > 300 mg/day vs no corticosteroids OR, 1.01; 95% CI 0.62, 1.64; *p* = 0.98; corticosteroids ≤ 300 mg/day vs no corticosteroids OR, 1.42; 95% CI 0.93, 2.17; *p* = 0.1). However, the initiation of corticosteroid in three days after ICU admission were associated with higher mortality in logistic regression model (OR, 4.49, 95% CI 1.17, 17.25, *p* = 0.025), Cox (HR, 3.89; 95% CI 1.94, 7.82; *p* < 0.001) and MSM (OR, 2.32, 95% CI 1.16, 4.65; *p* = 0.017). The association between corticosteroid therapy and higher 90-day mortality remained unchanged after the adjustment for clustering by site (data not shown).

### SARS-Cov-2 RNA clearance

Unadjusted analyses showed significant differences in the time of RNA clearance between patients receiving corticosteroid treatment and those who had not received corticosteroid treatment (10 days [IQR, 5–17.5] vs 7 days [1, 15], *p* = 0.049) (Table 1). Cox accounting for time-varying exposure showed no significant association between initiation of corticosteroid therapy and time to SARS-CoV-2 RNA clearance (HR, 1.10; 95% CI 0.36, 3.37; *p* = 0.86) (Table 3). Similarly, MSM analysis did not show significant association between corticosteroid therapy and SARS-CoV-2 RNA clearance (OR, 0.85; 95% CI 0.63, 1.17; *p* = 0.33). Sensitivity analysis also revealed that different doses of corticosteroid were not associated with SARS-CoV-2 RNA clearance. Although initiation of corticosteroids in 3 days after ICU admission promoted RNA clearance in Cox (HR, 0.26; 95% CI 0.13, 0.54; *p* < 0.001), this effect was no longer present after adjustment of time variables. In general, our analyses did not determine significant associations between corticosteroid treatment and SARS-CoV-2 RNA clearance.

## Discussion

Our study included 294 critically ill COVID-19 patients, with 62.2% patients having received corticosteroid therapy. Among those who received corticosteroid therapy, 96% were treated with methylprednisolone and 69.4% received corticosteroid treatment prior to ICU admission. After adjustment of baseline and time-dependent variables, an association between early corticosteroid treatment (≤ 3 days after ICU admission) and higher mortality was observed. Additionally, our study did not find an association between corticosteroid use and SARS-COV-2 RNA clearance.

Corticosteroids were widely used in the outbreaks of COVID-19 [15]. Severe COVID-19 is associated with a dysregulated host inflammatory response. The increasing number of infected epithelial cells and cell debrides trigger a massive cytokine release—the so-called ‘cytokine storm’ [16]. Histological examination of lungs from COVID-19 patients showed diffuse alveolar damage with cellular fibromyxoid exudates. Moreover, interstitial mononuclear inflammatory infiltrates, dominated by lymphocytes, were visible in lungs [17]. These findings provided a rationale for the use of corticosteroids in order to reduce inflammation. Consequently, the most severely affected COVID-19 patients (those with severe hypoxemic and higher SOFA scores) in our study were administrated corticosteroids (particularly methylprednisolone).

We found no significant association between corticosteroid treatment and 90-day mortality when subgroup analysis was not performed. Similarly, in Siemieniuk et al., short course treatment with methylprednisolone in hospitalized patients with COVID-19 was not associated with a reduction in mortality [4]. However, the RECOVERY trial by Huang et al. revealed that the use of dexamethasone resulted in lower 28-day mortality in patients randomized to receive invasive mechanical ventilation or oxygen alone, but not in patients who received no respiratory support [1]. Dexamethasone was selected in RECOVERY study, but methylprednisolone was the most commonly-used corticosteroid in our study. Differences in treatment regimens may lead to different anti-inflammatory effects and may have varying outcomes [18]. Additionally, the median duration of glucocorticoid therapy was 7 days in the RECOVERY trial and 9 days in our study. Long-term use of corticosteroids may cause adverse effects that obscure their effectiveness. Fadel et al. also showed that early short course of methylprednisolone reduced the requirement for mechanical ventilation and mortality [19]. However, since this study was a quasi-experimental study, the effects of multiple independent variables and their interactions on dependent variables cannot be analyzed. Additionally, concerns regarding statistical power should be considered in that study.

Notably, association of early initiation of corticosteroid therapy (≤ 3 days after ICU admission) and higher mortality was revealed when subgroup analysis was performed in our study, suggesting that patient selection had an influence on the outcome in COVID-19 patients who were initiated on corticosteroid treatment. For example, Siemieniuk et al. showed that corticosteroid treatment reduced mortality in hospitalized COVID-19 above the age of 60, but had no beneficial effects less severe patients [4]. Similarly, a benefit was not seen among less severe patients who did not require oxygen support in RECOVERY trial; there was no statistically significant upward trend in mortality [1]. Previous studies determined that the median time of illness onset to ARDS was 12 days for COVID-19 patients [20, 21]. In our study, the median time of illness onset to ICU admission was 11 days, with most corticosteroid therapy initiated prior to ICU admission. Therefore, we speculate that the adverse effects of corticosteroid therapy may be harmful for COVID-19 patients without ARDS. The anti-inflammatory role of steroids may be useful to mitigate the cytokine storm in COVID-19 patients with ARDS [22, 23].

Our results did not reveal that corticosteroid use was associated with SARS-CoV-2 RNA clearance. Several observational studies have reported that corticosteroid therapy was linked to persistent viral RNA shedding in patients with avian influenza A (H7N9), MERS, and SARS [24–26]. A retrospective study conducted by Xu et al. showed that prolonged viral RNA shedding in patients with COVID-19 was associated with corticosteroid treatment [27]. Herein, after adjusting for both immortal bias and indication bias, we found that corticosteroid usage was not associated with prolonged viral RNA shedding time. Accordingly, a retrospective COVID-19 study by Shi et al. indicated that corticosteroid had little effect on the duration of viral excretion [28]. The adverse effects of early initiation of corticosteroid therapy may be mediated by other side effects and require further investigation.

Our multi-center study analyzed the relationship between corticosteroid treatment and 90-day mortality of COVID-19 patients using logistic regression, Cox and MSM. Statistical power of our study is compelling and credible evidence for the effect of the corticosteroid treatment in COVID-19 patients was supplied. However, we also determined some limitations to our analyses. Firstly, the majority (127/183 or 69%) of the patients received corticosteroids prior to ICU admission, creating potential sampling bias, which may affect the evaluation of the role of corticosteroids. Secondly, we only focused on the effect of corticosteroids on mortality and RNA shedding. Arrhythmias, gastrointestinal bleeding, secondary infection and other corticosteroid-related side effects should be explored in future.

## Conclusions

Our findings demonstrate that early corticosteroid treatment (≤ 3 days after ICU admission) is associated with an increased 90-day mortality rate. We recommend that methylprednisolone should not be used or used with caution in the early stages of COVID-19.

## Supplementary Information

**Additional file 1.** Figure S1: The time of corticosteroid initiation before and after ICU admission. Day 0 means the day of ICU admission. Day -10 means 10 days before ICU admission.

**Additional file 2.** Clinical characteristic of patients with COVID-19 in the survivor and non-survivor.

## Data Availability

The datasets used and/or analyzed during the current study are available from the corresponding author on reasonable request.

## Abbreviations

COVID-19: Coronavirus disease 2019
MSM: Marginal structural modeling
SARS-CoV-2: Severe acute respiratory syndrome coronavirus 2
ICU: Intensive care unit
ARDS: Acute respiratory dyspnea syndrome
MERS: Middle east respiratory syndrome
SARS: Severe acute respiratory syndrome
APACHE II score: Acute Physiology and Chronic Health Evaluation II score
SOFA: Sequential Organ Failure Assessment
AKI: Acute kidney injury
KDIGO: Kidney disease: improving global outcomes
NAAT: Nucleic-acid amplification test
IQR: Interquartile range
PEEP: Positive end-expiratory pressure
