# Supplementary figures and images for: Corticosteroid therapy in critically ill patients with COVID-19: a multicenter, retrospective study
Source: Crit Care. 2020 Dec 18;24:698. doi: 10.1186/s13054-020-03429-w (PMC7747001; doi:10.1186/s13054-020-03429-w)

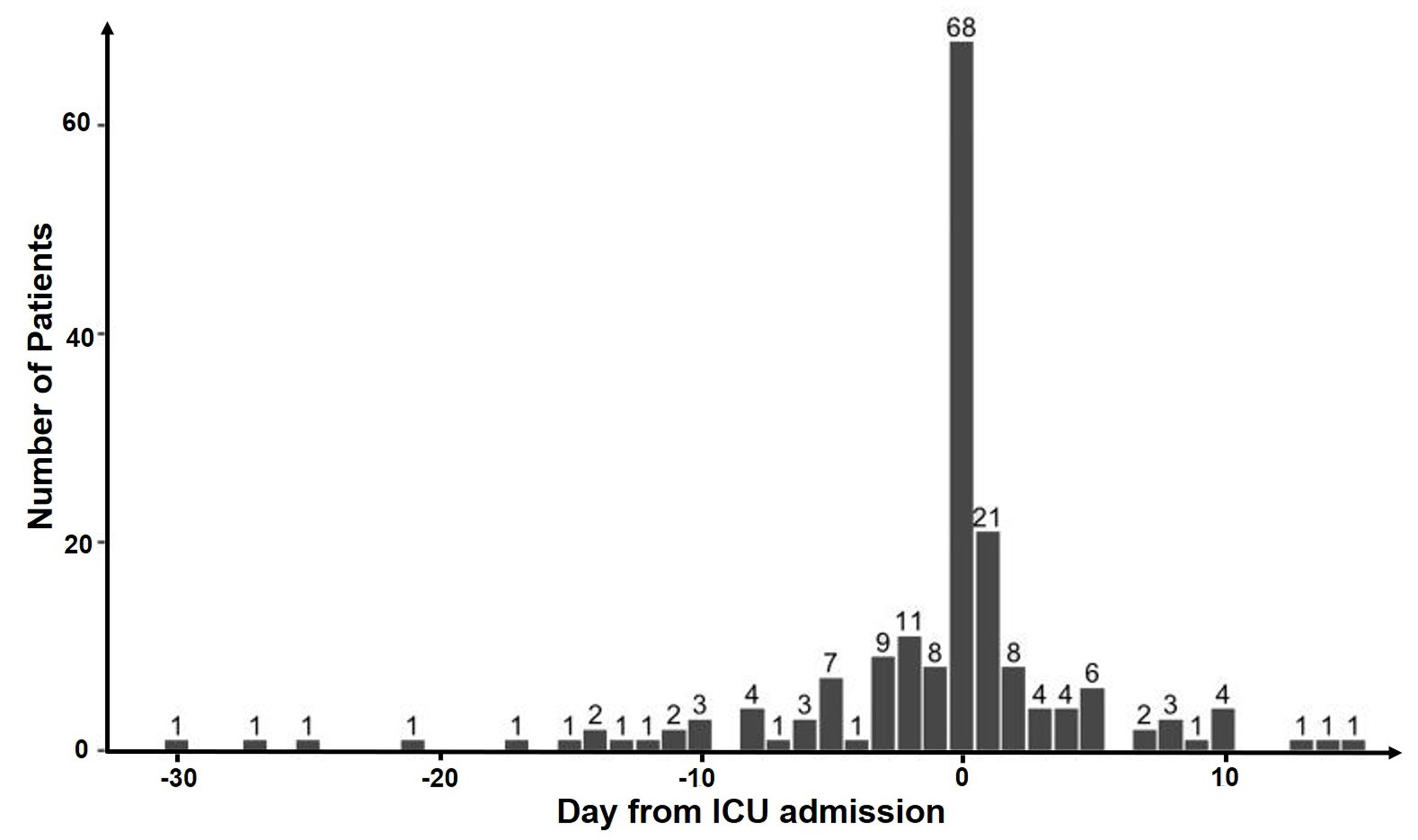

Supplement: Supplementary file 1 — Additional file 1. Figure S1: The time of corticosteroid initiation before and after ICU admission. Day 0 means the day of ICU admission. Day -10 means 10 days before ICU admission. [file 13054_2020_3429_MOESM1_ESM.jpg]
