# Supplementary material for: Corticosteroid therapy in critically ill patients with COVID-19: a multicenter, retrospective study
Source: Crit Care. 2020 Dec 18;24:698. doi: 10.1186/s13054-020-03429-w (PMC7747001; doi:10.1186/s13054-020-03429-w)
Supplement: Supplementary file 2 — Additional file 2. Clinical characteristic of patients with COVID-19 in the survivor and non-survivor. [file 13054_2020_3429_MOESM2_ESM.docx]

**Supplemental Table 1. Clinical characteristic of patients with COVID-19 in the survivor and non-survivor.**

|  | Total, n= 294 | Survivor, n= 148 | Non-Survivor , n= 146 | P value |
| --- | --- | --- | --- | --- |
| Age, year | 66 (56, 75) | 62 (52, 71) | 70 (62, 76.75) | < 0.001 |
| Male Sex | 197 (67) | 96 (65) | 101 (70) | 0.45 |
| Comorbidity |  |  |  |  |
| Hypertension, | 134 (46) | 61 (41) | 73 (50) | 0.16 |
| Diabetes | 80 (27) | 38 (26) | 42 (29) | 0.64 |
| Heart disease | 68 (23) | 27 (18) | 41 (28) | 0.063 |
| Chronic lung disease | 36 (12) | 18 (12) | 18 (12) | 1 |
| Cerebrovascular disease | 31 (11) | 14 (9) | 17 (12) | 0.68 |
| Immunosuppression | 19 (6) | 7 (5) | 12 (8) | 0.33 |
| PEEP, cmH2O | 9 (6, 10) | 8 (5, 10) | 10 (8, 10) | < 0.001 |
| PaO2/FiO2, mmHg | 138 (86.45, 204) | 172 (112, 255) | 110 (75.25, 165.3) | < 0.001 |
| Platequ pressure, cmH2O | 25 (21, 26) | 25 (20, 26) | 25 (22, 27.25) | 0.24 |
| Mean arterial pressure, mmHg | 90 (80, 97) | 90 (82, 98) | 89 (78, 96) | 0.11 |
| Creatinine, μmol/L | 76 (61.7, 101.22) | 67.85 (56.1, 85.45) | 86.5 (65.97, 124.75) | < 0.001 |
| Elevated TnI, | 95 (41) | 30 (27) | 65 (54) | < 0.001 |
| Bilirubin, μmol/L | 22.2 (13.2, 24) | 22 (12.93, 23) | 23 (14.07, 24) | 0.017 |
| Platelet count, ×la^9^/L | 158 (114, 207.25) | 186 (136.25, 236.75) | 145 (98.5, 175.75) | < 0.001 |
| Lymphocyte percentage | 6.24 (2.6, 11.31) | 8.1 (2.76, 14.12) | 5.09 (2.36, 8.49) | 0.011 |
| APACHEII score | 14.5 (11, 18) | 12 (9, 16) | 17 (13, 22) | < 0.001 |
| SOFA score | 4 (3, 5.75) | 3 (2, 4) | 5 (3, 7) | < 0.001 |
| Complication |  |  |  |  |
| Shock, | 121 (42) | 20 (14) | 101 (71) | < 0.001 |
| ARDS, | 214 (74) | 89 (60) | 125 (87) | < 0.001 |
| AKI, | 90 (31) | 12 (8) | 78 (55) | < 0.001 |
| Secondary infection, | 44 (15) | 16 (11) | 28 (19) | 0.065 |
| Respiratory support received |  |  |  |  |
| Oxygen only | 64 (21) | 35 (18.2) | 29 (26.1) | 0.159 |
| Mechanical ventilation | 230(78.2) | 148(80.8) | 82(73.9) | 0.031 |
| Prone | 46 (16) | 15 (10) | 31 (21) | 0.01 |
| ECMO | 21 (7) | 8 (5) | 13 (9) | 0.249 |
| Tracheotomy | 23 (8) | 11 (7) | 12 (8) | 0.802 |
| Requirement of CRRT | 40 (14) | 6 (4) | 34 (23) | <0.001 |
| Symptom onset to hospital admission, day | 7(4-10) | 7(4-10) | 7(3-14) | 0.13 |
| Symptom onset to ICU admission, day | 11 (7, 17) | 11 (6, 15) | 12 (7.25, 18) | 0.26 |
| Duration of ventilation, day | 7 (0, 13) | 3 (0, 10) | 9 (3.25, 15.75) | < 0.001 |
| ICU length of stay, day | 14 (7, 23) | 15.5 (9, 24) | 12 (6, 20) | 0.004 |
| Hospital length of stay, day | 20 (12, 32) | 27.5 (16, 38.25) | 14.5 (9, 24) | < 0.001 |
| Time to RNA clearance among 90-d survivors, day | 9 (3, 17) | 10 (6, 18) | 4 (0, 10.5) | < 0.001 |

Abbreviations: PEEP =positive end-expiratory pressure; Q=quartile; SOFA= Sequential Organ Failure Assessment; APACHEII = acute physiology and chronic health evaluation; Tnl = Troponin I; ECMO=extracorporeal membrane oxygenation; ARDS= adult respiratory distress syndrome; AKI=acute kidney injury;

Immunosuppression was defined as human immunodeficiency virus infection, malignancy, chemotherapy, organ transplantation. Laboratory and ventilator parameters on day 1 of ICU admission were presented. Data presented as n (%) or median (Q1–Q3).
